# Supplementary figures and images for: Effect of Slightly Acidic Electrolyzed Water on Growth, Diarrhea and Intestinal Bacteria of Newly Weaned Piglets
Source: Genes (Basel). 2023 Jul 4;14(7):1398. doi: 10.3390/genes14071398 (PMC10378913; doi:10.3390/genes14071398)

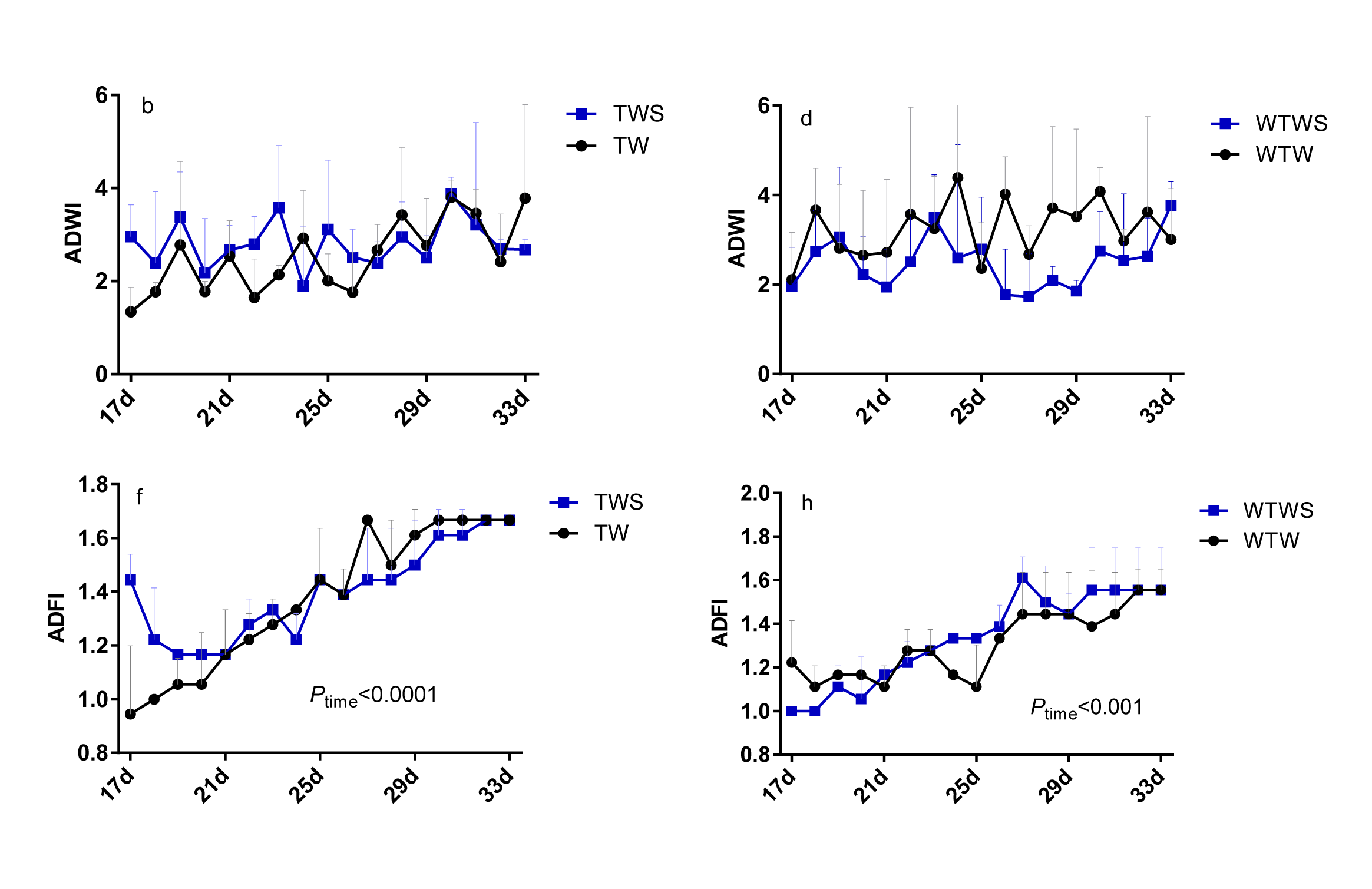

Supplement: Supplementary file 1 [file genes-14-01398-s001.zip › genes-2479494-supplementary-Figure S1.png]
